# Mass spectrometry peptidomics data from infected and uninfected porcine wounds

**DOI:** 10.1038/s41597-025-05842-8

**Published:** 2025-09-02

**Authors:** Fredrik Forsberg, Sven Kjellström, Jitka Petrlova, Manoj Puthia, Artur Schmidtchen, Johan Malmström, Erik Hartman

**Affiliations:** 1 Division of Dermatology and Venereology, Department of Clinical Sciences, Lund University, Lund, Sweden; 2 Division of Extracellular Signaling and Cell Fate, Department of Experimental Medical Sciences, Lund University, Lund, Sweden; 3 Division of Mass Spectrometry, Department of Clinical Sciences, Lund University, Lund, Sweden; 4 Department of Biomedical Science, Faculty of Health and Society, Malmö University, 205 06 Malmö, Sweden; 5 Biofilms Research Centre for Biointerfaces, Malmö University, 205 06 Malmö, Sweden; 6 Division of Infection Medicine, Department of Clinical Sciences Lund, Faculty of Medicine, Lund University, Lund, Sweden

**Keywords:** Biomarkers, Pathogens, Infection

## Abstract

Recently, mass spectrometry based peptidomics studies have proven useful in the identification of biomarkers and bioactive peptide-based therapeutics. Here, we present a dataset comprised of temporal wound fluid peptidomics data from highly defined porcine models. Wound fluids from porcine wounds infected with *Staphylococcus aureus* and *Pseudomonas aeruginosa*, and uninfected controls, were sampled at different timepoints of the infection. Peptides were extracted from the samples, followed by liquid chromatography tandem mass spectrometry analysis in data dependent acquisition mode. The resulting spectra and searched files have been deposited to online repositories and made easily accessible to enable further investigations of the infected and uninfected wound fluid peptidome.

## Background & Summary

Peptides are short sequences of amino acids which are naturally produced in organisms, both through the translation of mRNA but importantly also through the degradation of proteins. These peptides play important roles in different biological systems, such as messengers in signalling pathways^1^ and by providing antimicrobial properties during infection^2–4^. Peptidomics is the study of large sets of peptides from biological samples and has proven to be pivotal in the characterization of peptides and protein degradation under different physiological and pathological conditions as well as in the search for therapeutic peptides. Identification and quantification of peptides is necessary to conduct peptidomic analyses. Commonly, liquid chromatographic (LC) separation followed by mass spectrometry (MS) analysis is used for this purpose^5^.

During a wound infection, a combination of host and pathogen derived proteases create an environment with increased proteolytic activity. The host uses proteases to remodel tissue and fight the invasion of bacteria, whereas bacterial proteases may promote colonization^6–9^. By investigating the resulting peptidome during different wound infection conditions, further understanding of this environment and mechanism could be gained.

Chronic wounds pose a large economic burden on society and life quality burden for patients. The prevalence of these wounds is likely to increase with an aging population and more life-style related diseases^10^. Further, pathogens such as *Staphylococcus aureus* and *Pseudomonas aeruginosa* are two of the most prevalent bacteria in infected wounds^11^. They are considered of high and critical priority respectively by the World Health Organization due to their developed resistance against current antibiotics^12^. Therefore, identifying novel means of diagnosing and treating such wounds is of extreme importance.

**Fig. 1:**
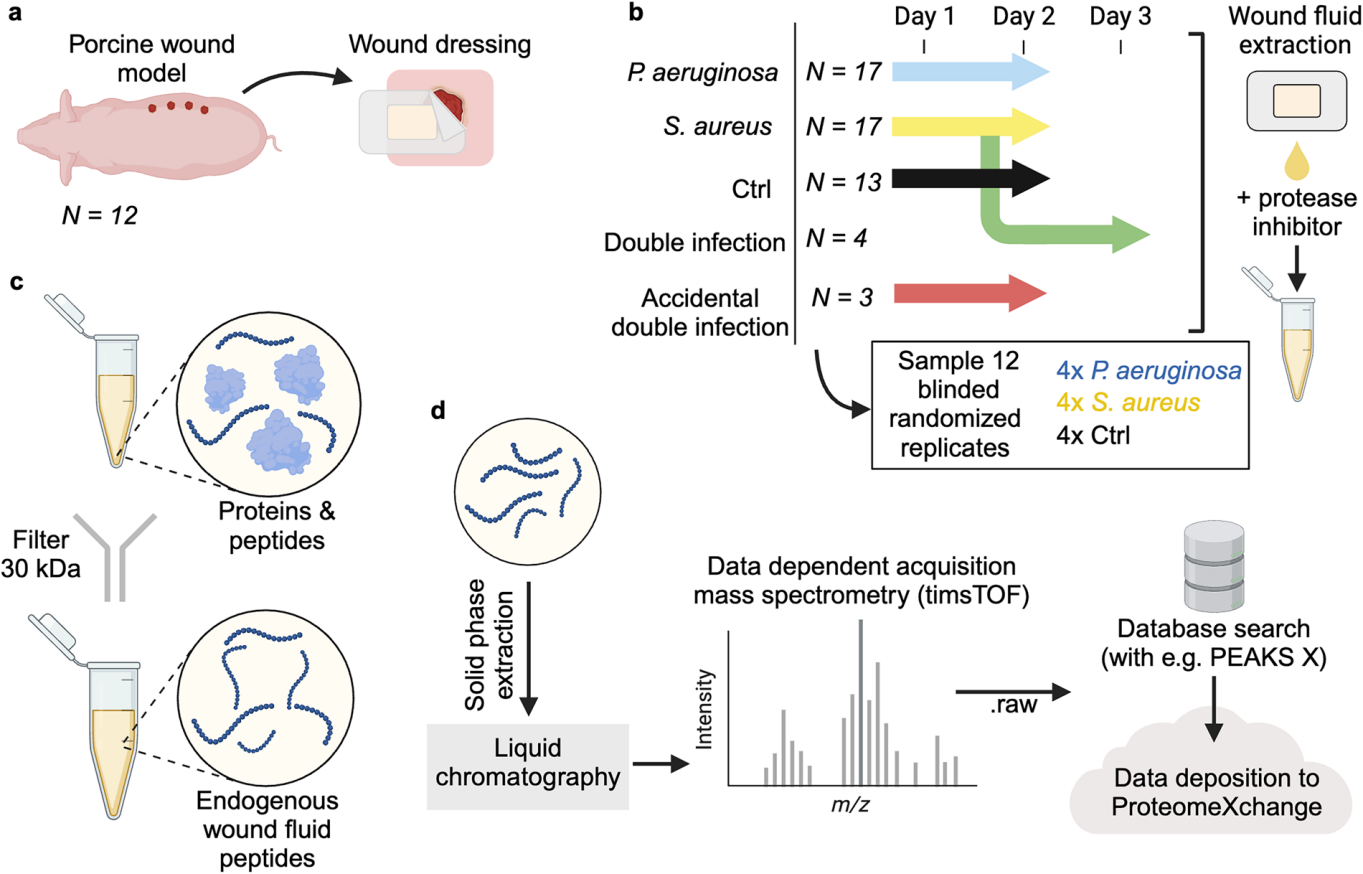
Schematic illustration of the samples, the sample preparation, and analysis protocol. (**a**) Wounds were generated onto pigs and overlaid with polyurethane dressings. The dressings absorbed the fluids from the wound. (**b**) Dressings were collected at every 24 hours over a 2–3-day period. The wound fluids were extracted from the dressings and protease inhibitor was added. (**c**) Wound fluids were filtered to separate the peptides from the proteins and other larger molecules found in the samples. (**d**) Filtered peptides were purified using solid phase extraction, before liquid chromatography-mass spectrometry analysis in DDA mode. The results were then searched with PEAKS X and both the searched and raw files were uploaded to ProteomeXchange.

We previously carried out a study to identify differences in the peptidomic landscape of wound fluids depending on the presence and type of pathogen, while also employing a newly developed analysis algorithm with the potential to remove large amounts of redundancy in peptidomic datasets^13^. This was achieved by generating LC-MS/MS data from wound fluids derived from highly defined infected porcine wounds (Fig. 1). Here, we present descriptions and access to the datasets. The extensive nature of the peptidomic data yields many opportunities to analyze it using different methods to gain novel insights about the mechanisms underlying protein degradation in infected wounds.

## Methods

### Ethics statement

The animal experiments were conducted according to Swedish Animal Welfare Act SFS 1988:534 and were approved by the Animal Ethics Committee of Malmö/Lund, Sweden, under permit number M131-16.

### Pig acute wound fluid collection

Partial thickness wounds were induced to 14 Göttingen minipigs, which were then either infected with *S. aureus* or *P. aeruginosa*, both, or kept uninfected as controls. Polyurethane dressings were used to dress the wounds and were changed after 24 and 48 hours (Fig. 1a). After removal, the dressings were soaked in 10 mM Tris, and the fluids were extracted using a syringe. A subset of the *S. aureus* infected wounds was accidentally cross contaminated with *P. aeruginosa*. Another set of the *S. aureus* infected wounds was infected with *P. aeruginosa* after 24 hours, and these dressings were also collected and extracted 72 hours after the initial *S. aureus* infection. Extracted fluids were then supplemented with Halt Protease Inhibitor Cocktail (Thermo Fisher Scientific, USA) and kept at −80 °C until further use (Fig. 1b).

### Wound fluid peptide extraction

From the collected wound fluid, 500 μg of protein (determined with Pierce BCA Protein Assay Kit (Thermo Fisher Scientific, USA) as per provided instructions) was diluted to 100 μl with 10 mM Tris at pH 7.4. To this, 300 μl of 8 M urea diluted in 10 mM Tris at pH 7.4, supplemented with 0.067% RapiGest SF (Waters, USA) was added followed by an incubation at room temperature (RT) for 30 minutes. Microcon Centrifugal Filters, NMWCO 30 kDa (Merck, Germany) units were wet with 100 μl 6 M urea in 10 mM Tris at pH 7.4 and centrifuged at 10000 RCF for 15 minutes at RT. The wound fluid samples were subsequently added to the centrifugal filters, and centrifuged at 10000 RCF for 30 minutes at RT. Finally, another 100 μl of 6 M urea in 10 mM Tris at pH 7.4 was centrifuged through the filter units at 10000 RCF for 5 minutes at RT and the filtrate was stored at −20 °C (Fig. 1c).

### Acidification and solid phase extraction

The extracted peptide samples were acidified by adding 1 μl 100% formic acid (FA) to 60 μl of each sample. This was followed by adding 100 μl 100% acetonitrile (ACN) + 0.1% FA to UltraMicro Spin Columns (The Nest Group, USA) which were centrifuged at 800 RCF for 1 minute at room temperature. All further centrifugation steps in this section were performed this way. Next, 100 μl 2% ACN + 0.1% trifluoroacetic acid (TFA) was centrifuged through the columns, twice, before adding the samples and performing an additional centrifugal step. Lastly, 100 μl 70% ACN + 0.1% TFA was centrifuged through the columns to elute the sample which was then dried in an Eppendorf Concentrator plus (Eppendorf, Germany) (Fig. 1d).

### LC-MS/MS

The dried peptide samples were then dissolved in 30 μl 2% ACN + 0.1% FA, before being loaded onto Evotip Pure columns (Evosep, Denmark) according to the manufacturer’s instructions, apart from that the samples were not dissolved in 20 µl 0.1% FA before loading. The samples were analyzed by LC/MS-MS on an Evosep One LC (Evosep, Denmark) coupled with a timsTOF Pro mass spectrometer (Bruker, USA). The LC used a EV1137 Performance Column - 15 cm × 150 µm, with 1.5 µm ReproSil-Pur C18 beads (Evosep, Denmark). The accompanying 30 samples per day program was used for separation. The MS data was acquired using the DDA PASEF mode, with 10 PASEF scans every acquisition cycle. Accumulation and ramp times were set to 100 ms, precursors with a +1 charge were ignored, and target intensity was set to 20000, with dynamic exclusion active, at 0.4 min. Isolation width was set to 2 at 700 Th and 3 at 800 Th (Fig. 1d).

### Database search

The data from the LC-MS/MS runs were searched with PEAKS X. UniProtKB reviewed (Swiss-Prot) protein list of pig (*Sus scrofa*, organism_id:9823) proteins was used as a database, but with fibrinogen alpha chain (FIBA_PIG) and fibrinogen beta chain (FIBB_PIG) being changed to the UniProt KB unreviewed (TrEMBL) versions F1RX36_PIG and F1RX37_PIG. The list was downloaded May 11^th^, 2023. Data refinement was set to merge scans and correct precursor based on mass and charge states with charges between 1 and 4. It was also set to associate features between 2 and 8. Precursor tolerance was set to 20.0 ppm using monoisotopic mass and fragment tolerance was set to 0.03 Da. 1 modification per peptide was allowed with methionine oxidation being the only possible modification. Results were filtered at 1% FDR with ≥1 unique peptide for each protein. FDR was set to be estimated with decoy-function (Fig. 1d).

## Data Records

Both the raw mass spectrometry data (.d folders generated by Bruker Compass) as well as the database search of the data (.mgf, .mzid.gz and .csv generated by PEAKS X) have been uploaded to ProteomeXchange as a part of the public dataset PXD048892 along with a design file for sample identification (.csv)^14^. Additionally, similar files from the blinded re-run of samples have been uploaded to ProteomeXchange under the identifier PXD055074^15^. All data has also been uploaded to GitHub, alongside code to easily load and analyze the data.

## Technical Validation

**Fig. 2:**
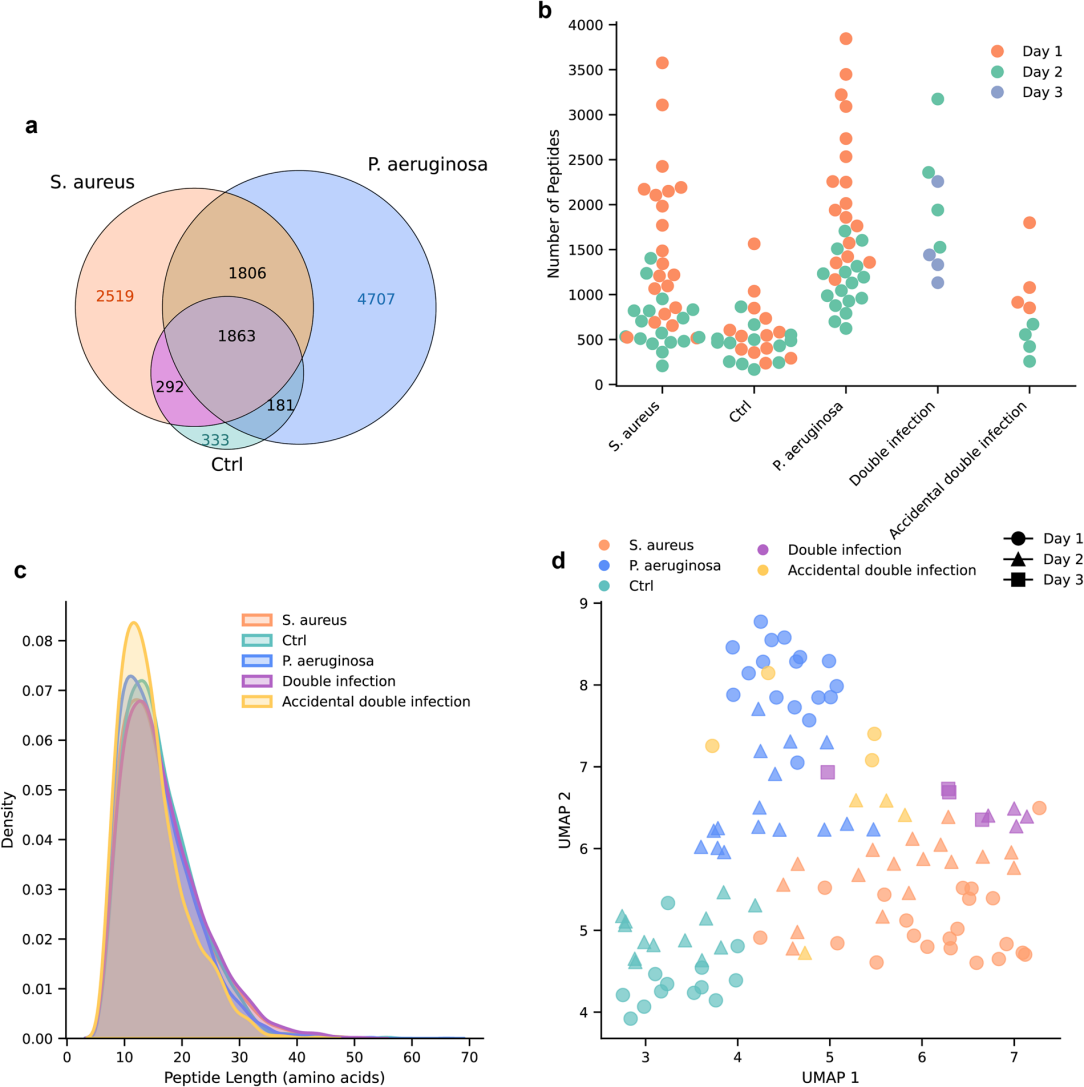
Characteristics of samples. (**a**) Venn diagram showing the distribution of unique peptides between the samples from wounds infected with S. aureus, P. aeruginosa and the uninfected controls. (**b**) The number of unique peptides observed in each sample. (**c**) Kernel density estimates of peptide length weighted by the log_2_ peptide intensity. Separated on sample type. **d** Uniform Manifold Approximation and Projection (UMAP) dimensionality reduction based on the log_2_-transformed intensities of all peptides in each sample.

To characterize the dataset, general characteristics of the different groups are summarized in Fig. 2. Unique peptide overlap was summarized, with a higher number of unique peptides identified in the *S. aureus* (2519) and *P. aeruginosa* (4707) groups compared to the control group (333), while the number of peptides shared by all sample groups was 1863 (Fig. 2a). The number of identified peptides decreased over time (Fig. 2b). The mean length distribution weighted by the log_2_ intensities were similar for all sample types (Fig. 2c). The log_2_ intensities were scaled to a mean of 0 and a unit variance. Thereafter, the dimensionality of the data was reduced using Uniform Manifold Approximation Projection (UMAP). Default settings were used as per the umap-learn python package (Fig. 2d). The data cluster based on infection type and sampling day.

**Fig. 3:**
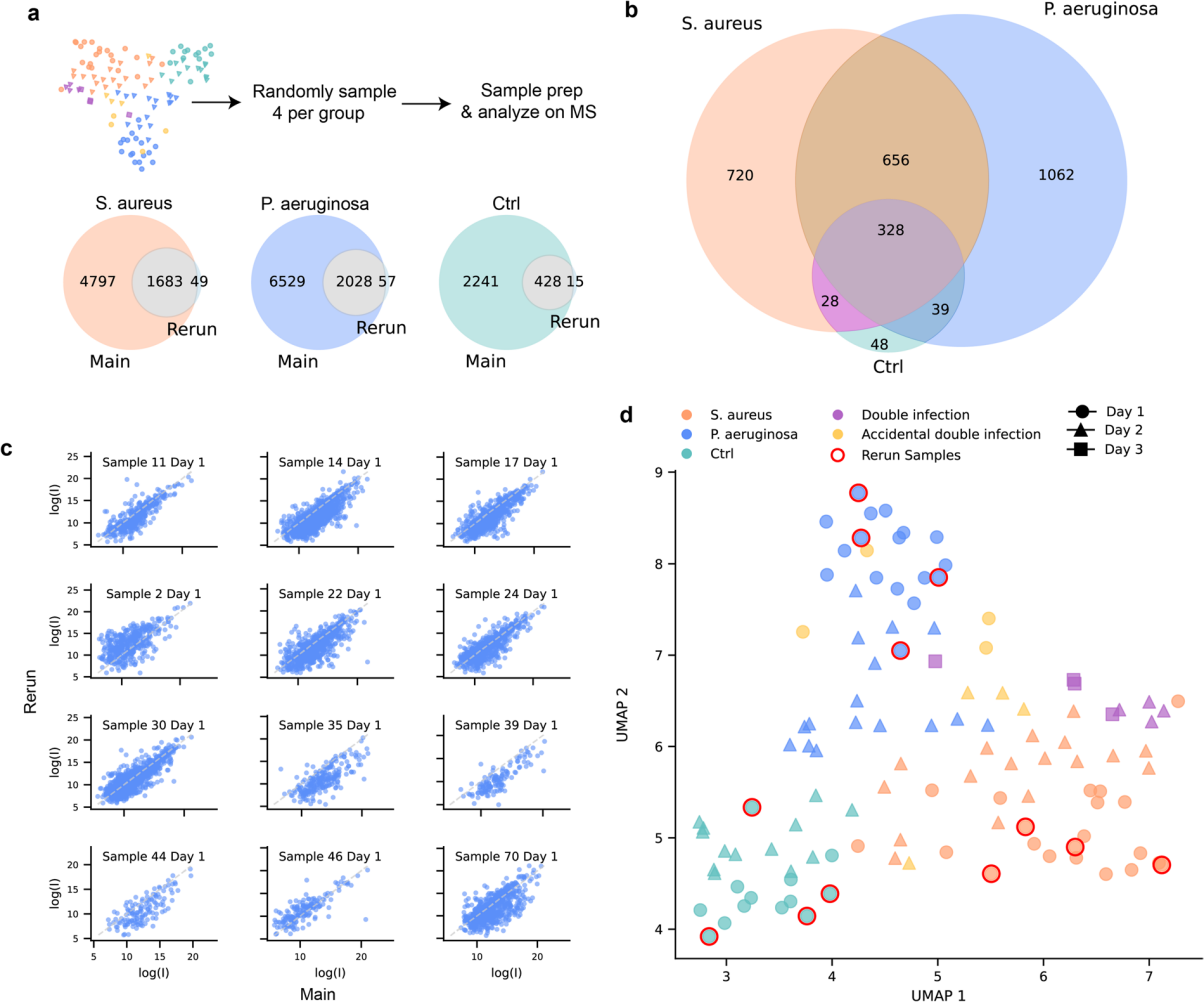
Characterization and comparison with blinded replicates. (**a**) 4 samples were randomly and blindly sampled from the day 1 samples of each sample type and the analysis was replicated. The Venn diagrams show the unique peptides observed in sample groups compared to the same groups during the blinded rerun. (**b**) Venn diagram comparing the number of unique peptides in the blinded samples. (**c**) Scatter plots showing the log_2_-intensity of the common peptides present in the blinded rerun and the original corresponding samples. (**d**) Uniform Manifold Approximation and Projection (UMAP) dimensionality reduction based on the log_2_-transformed intensities of all peptides in each sample with blinded rerun samples added. The rerun samples are highlighted with a red outline.

To validate that our peptide extraction and mass spectrometry methods are reproducible, 4 wound fluid samples from day 1 and each of the groups *S. aureus*, *P. aeruginosa*, and control were randomly selected, and the peptides were extracted as per the protocol described previously starting at the “Wound fluid peptide extraction” step described above (Fig. 1c). The samples were blinded for the entire sample preparation and analysis workflows. The peptides were analyzed by LC-MS/MS as previously, but using a timsTOF HT (Bruker, USA). The unique peptides identified in each sample group corresponded well with the samples analyzed previously (Fig. 3a). Relative number of unique peptides are similar for the blinded re-analyzed samples compared to the original (Fig. 3b). The log_2_ intensities of peptides found in both the original samples and the blinded rerun were similar (Fig. 3c). Lastly, the samples cluster together with the other samples from the same group and day when reducing dimensionality using UMAP (Fig. 3d), showing that the method is robust and replicable.

## Usage Notes

The data deposited online was supplied as both raw output files and result files searched with PEAKS X. The raw files can be used for searching the data with different softwares or parameters than the one presented in this study. Alternatively, the searched data can be accessed directly. Instructions for loading and processing the data can be found in the accompanying GitHub repository.

There are many ways to analyze the data in the resulting output files. MS intensities follow a lognormal distribution, so for comparative analyses the log of the intensities is computed prior to assuming normal distributions. The data contains missing values that can be reduced in different ways, e.g. through imputation. Further, to remove technical bias effects, normalization of the peptide intensities is commonly applied.

## Data Availability

This code is accompanied by a GitHub repository: https://github.com/Fredan97/porcine-peptidomics-data. The repository contains code for easily loading and processing the data and recreating the figures alongside the raw and processed files.
